# Effects of Calcium-Oxide-Modified Biochar on the Anaerobic Digestion of Vacuum Blackwater

**DOI:** 10.3390/molecules30020215

**Published:** 2025-01-07

**Authors:** Ping Fa Chiang, Teng Ling Zhang, Abdulmoseen Segun Giwa, Ndungutse Jean Maurice, Mugabekazi Joie Claire, Nasir Ali, Ehtisham Shafique, Mohammadtaghi Vakili

**Affiliations:** 1 School of Economics and Management, Nanchang Institute of Science and Technology, Nanchang 330100, China; raymond.chiang9278@hotmail.com; 2 School of Education, Nanchang Institute of Science and Technology, Nanchang 330108, China; ozhangtenglingk@163.com; 3 School of Civil and Environmental Engineering, Nanchang Institute of Science and Technology, Nanchang 330108, China; 4 Institute of Environmental Science, Shanxi University, Taiyuan 030006, China; 5 Faculty of Education, Southwest University, Chongqing 400715, China; mugabekazijoie@gmail.com; 6 Department of Biological and Health Sciences, Pak-Austria Fachhochschule: Institute of Applied Sciences and Technology, Khanpur Road, Haripur 22621, Pakistan; ehtishamshafique94@gmail.com; 7 Orlen Unicre a.s., Revolucňí 1521/84, 400 01 Ústí nad Labem, Czech Republic; mohammadtaghi.vakili@orlenunicre.cz

**Keywords:** vacuum blackwater, calcium oxide-modified biochar, anaerobic digestion, integrated methods, bioenergy recovery

## Abstract

The increasing global population and urbanization have led to significant challenges in waste management, particularly concerning vacuum blackwater (VBW), which is the wastewater generated from vacuum toilets. Traditional treatment methods, such as landfilling and composting, often fall short in terms of efficiency and sustainability. Anaerobic digestion (AD) has emerged as a promising alternative, offering benefits such as biogas production and digestate generation. However, the performance of AD can be influenced by various factors, including the composition of the feedstock, pH levels, and the presence of inhibitors. This review investigates the effects of calcium oxide (CaO)-modified biochar (BC) as an additive in AD of VBW. Modifying BC with CaO enhances its alkalinity, nutrient retention, and adsorption capacity, creating a more favorable environment for microorganisms and promoting biogas production, which serves as a valuable source of heat, fuel and electricity. Additionally, the digestate can be processed through plasma pyrolysis to ensure the complete destruction of pathogens while promoting resource utilization. Plasma pyrolysis operates at extremely high temperatures, effectively sterilizing the digestate and eliminating both pathogens and harmful contaminants. This process not only guarantees the safety of the end products, but also transforms organic materials into valuable outputs such as syngas and slag. The syngas produced is a versatile energy carrier that can be utilized as a source of hydrogen, electricity, and heat, making it a valuable resource for various applications, including fuel cells and power generation. Furthermore, the slag has potential for reuse as an additive in the AD process or as a biofertilizer to enhance soil properties. This study aims to provide insights into the benefits of using modified BC as a co-substrate in AD systems. The findings will contribute to the development of more sustainable and efficient waste management strategies, addressing the challenges associated with VBW treatment while promoting renewable energy production.

## 1. Introduction

The treatment of vacuum blackwater (VBW), a complex mixture of human waste, water, and other organic materials collected from vacuum toilets, presents significant challenges due to its unique composition. This includes high organic loads and a notable presence of pathogens, such as eggs of intestinal parasites like *Ascaris, Trichuris*, and *Toxocara*, as well as bacteria from the genera *Salmonella* and *Escherichia coli* [1]. These pathogens pose serious health risks, leading to infections and diseases in humans and animals, and they also negatively impact the environment [2,3]. Moreover, the organic matter in VBW can contribute to the depletion of oxygen in aquatic ecosystems, resulting in harmful effects, including fish kills and loss of biodiversity [4]. Currently, the generation of VBW has significantly increased due to the development of transportation systems, including airplanes, trains, and ships. For instance, global air travel has reached approximately 4.5 billion passengers annually [5]. In China, rail transport statistics indicate that around 1.3 billion passengers generate more than 5.3 million tons of VBW each year [6]. Anaerobic digestion (AD) has emerged as a promising technology for treating such waste, converting organic matter into biogas and digestate. Research by Arifan et al. [7] demonstrated that introducing livestock manure and liquid tofu waste into the AD process resulted in significant reductions in biochemical oxygen demand (BOD) and chemical oxygen demand (COD), achieving removal rates of over 95% and 98%, respectively. Additionally, AD promotes the recovery of essential nutrients such as nitrogen (N), phosphorus (P), potassium (K), and other valuable chemical compounds, which can be reused as organic fertilizers [8]. However, the efficiency of AD can be hindered by various factors, including the presence of toxic compounds, pH levels, and recalcitrant organic materials that resist degradation. Furthermore, Rajat et al. [9] reported that the digestate produced from AD can contain harmful pathogens, potentially leading to secondary pollution if not managed properly. Therefore, there is a critical need for environmentally sustainable treatment methods to enhance bioenergy production and promote resource recovery. Addressing these challenges is essential to ensure that the process maximizes biogas production while safely managing digestate, contributing to a more sustainable waste management system.

Biochar is a valuable by-product obtained from the pyrolysis process, which involves the thermal decomposition of organic materials in the absence of oxygen [10]. Recently, the use of BC as an additive in AD has shown significant potential for enhancing overall performance. According to Parmila et al. [11], BC can facilitate the degradation of organic matter by providing a stable structure that supports microbial communities, thereby increasing their activity and efficiency. Additionally, it aids in pH regulation and reduces inhibitors that can impede the digestion process, such as volatile fatty acids (VFAs) and ammonia. Ding et al. [12] reported that the porous structure of BC offers a habitat for anaerobic microorganisms, promoting microbial colonization and growth. Viaene et al. [13] found that mixing BC with digestate can decrease soil carbon dioxide emissions by up to 33% while enhancing the nutrient profile of the digestate. Moreover, Lee et al. [14] demonstrated that the use of BC can help mitigate odors associated with AD by adsorbing volatile organic compounds that contribute to unpleasant smells, thereby improving the overall environmental impact of AD facilities. However, conventional BC has several limitations, including poor adsorption capacity, inadequate surface charge properties, and insufficient pH regulation, which can hinder its effectiveness in various applications, particularly in AD treatment. These deficiencies also make it less capable of removing inhibitors that can adversely affect biogas production. Consequently, there is growing interest in modifying BC through various treatments to enhance its properties and make it a more suitable additive for improving the efficiency of the AD process. Several studies have focused on modifying BC to improve its selectivity, increase functional groups, enhance specific surface area, and boost stability. For instance, Chiang et al. [15] modified BC with polyvinyl alcohol and chitosan to enhance its adsorption capacity toward heavy metals and dyes in AD. Additionally, Zhang et al. [16] functionalized BC with iron-manganese compounds and employed it as an additive in AD. Wang et al. [17] also investigated the preparation, characterization, and modification of BC for environmental applications.

This review advocates the use of CaO-modified BC as an additive in AD to enhance the breakdown of VBW. The modified BC can facilitate electron transfer, regulate pH levels, and eliminate inhibitors, all of which contribute to increased biogas production and improved nutrient recovery. Feeding the digestate produced from AD into plasma pyrolysis generates syngas and slag, significantly reducing the volume of digestate while immobilizing harmful pathogens and toxic substances. The syngas and biogas produced from plasma pyrolysis and AD, respectively, can be utilized for various applications, including electricity generation, fuel production, and heat energy. This dual bioenergy recovery not only maximizes the value derived from VBW, but also contributes to sustainable energy solutions. Furthermore, the slag generated from plasma pyrolysis can be repurposed as a biofertilizer, enhancing soil health and fertility or as an additive in the AD process. This material recycling supports a circular economy, transforming waste into valuable resources and promoting environmental sustainability while improving agricultural productivity.

## 2. Assessment of Biochar

Biochar is a carbon-rich material produced through the pyrolysis of organic biomass, a process that involves heating the material in the absence of oxygen [18]. In contrast to torrefaction, which takes place at temperatures between 200 and 300 °C [19], pyrolysis occurs at much higher temperatures, generally ranging from 300 to 700 °C [10]. This process results in the decomposition of organic materials into various products, such as BC, bio-oil, and syngas [20]. The physical and chemical properties of BC are greatly affected by the working conditions of the pyrolysis process, including feedstock type, temperature, and residence time [21]. Different feedstocks, such as wood, agricultural residues, and municipal waste, possess varying chemical compositions and structures, which directly affect the characteristics of the resulting BC [10,22]. Temperature plays a crucial role; higher pyrolysis temperatures generally lead to increased carbon content and stability, while lower temperatures can result in higher yields of volatile compounds and bio-oil. Additionally, residence time influences the degree of decomposition and transformation of organic materials. Longer residence times typically enhance carbonization and alter the surface area and porosity of BC, thereby improving its adsorption capacity for nutrients and contaminants [23,24]. The fundamental pyrolysis process, mass balance, and energy equilibrium were well shown in Equations (1), (2), and (3), respectively.

Biomass + Pyrolysis (300–700) °C → BC + Bio-oil + Syngas(1)

M_Biomass_ = M_BC_ + M_Bio-oil_ + M_Syngas_ + M_Loss_ (2)

E_Biomass_ + E_Heat_ = E_BC_ + E_Bio-oil_ + E_Syngas_ + E_Loss_ (3)

where M is the ratio of total mass production to whole mass intake, while E_Biomass_ and E_Heat_ are the biomass energy and heat energy needed for the pyrolysis mechanism, respectively [20].

The production of BC has garnered significant interest due to its multifaceted benefits. As a soil amendment, BC enhances soil fertility, improves water retention, and promotes microbial activity, resulting in healthier and more productive agricultural systems [8]. Additionally, it plays a crucial role in carbon sequestration, helping to mitigate climate change by storing carbon that would otherwise be released into the atmosphere [13]. Its use also contributes to waste management by providing a sustainable method for recycling organic waste materials, which not only reduces landfill usage, but also decreases greenhouse gas emissions associated with waste decomposition. Beyond these applications, BC can be utilized in the water purification processes, where its porous structure helps remove contaminants and improve water quality. Furthermore, Parmila et al. [11] reported that BC has potential applications in other treatment processes such as AD, where it can serve as an additive to enhance the efficiency of biogas production. Physiochemical properties of BC were presented in Table 1.

## 3. Modification of Biochar with Calcium Oxide

**Figure 1:**
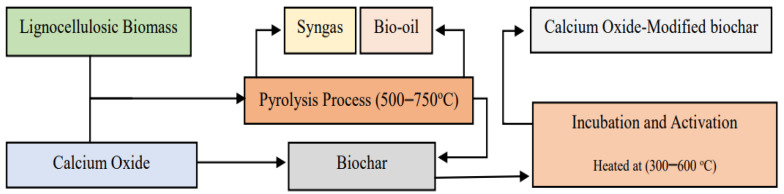
Simple route of biochar production and modification.

Modification of BC with CaO represents a promising approach to enhance its properties and functionalities for various applications, particularly waste management and environmental remediation. Biochar possesses inherent qualities such as high surface area and porosity, making it an effective adsorbent for contaminants [12,26]. However, its efficacy can be significantly improved through chemical modification [14,15]. The incorporation of CaO not only increases the alkalinity of BC, but also enhances its adsorption capacity and reactivity [27]. This modification can facilitate better interactions with pollutants and pathogens, making modified BC particularly valuable in processes like AD, where it can improve biogas production and nutrient recovery [28]. Furthermore, the alkaline nature of CaO can help mitigate acidity issues and stabilize nutrients, promoting a healthier microbial environment. As such, the modification of biochar with calcium oxide opens new avenues for optimizing its use in sustainable waste management practices and addressing environmental challenges. Figure 1 presents the production route and modification of BC.

As shown in Figure 1, the generation of CaO-modified BC includes several processes: BC production begins with the collection and preparation of organic biomass. Thereafter, biomass can be processed, typically by shredding or drying, to ensure uniformity and optimal conditions for pyrolysis. In the pyrolysis process, the prepared biomass is heated in a reactor at high temperatures around (500–700) °C in the absence of oxygen [22]. This thermal decomposition converts the biomass into three main products, such as BC, syngas, and bio-oil [20]. Once the BC is produced, it can be modified to enhance its properties for further use. The first common method involves mixing the BC with CaO, which can be performed through dry or wet mixing techniques. The mixture is then subjected to further heating, often in the range of (300–600) °C, to activate the CaO and allow better interaction between the BC and CaO. Thermal activation aids in the creation of calcium carbonate (CaCO_3_) and other calcium-based compounds during the modification process, further improving the alkalinity and buffering capacity of the BC [27,29]. Moreover, the heating process helps eliminate any remaining moisture and volatile compounds in the BC, which might otherwise impede the interaction between the two materials.

The second method is mixing raw biomass with CaO, and then introducing it into the pyrolysis process. Based on Qing et al. [27], CaO promotes the pyrolysis process, as it aids in the dehydration of biomass by absorbing moisture, which can enhance the devolatilization process. This leads to a more efficient conversion of biomass into gaseous and liquid products during pyrolysis. The use of CaO in pyrolysis can also facilitate the capture of carbon dioxide, forming CaCO_3_ during the process [29]. This not only helps in reducing greenhouse gas emissions, but also contributes to the overall sustainability of the pyrolysis process. Moreover, it promotes the breakdown of larger organic molecules, which can help in the reduction of tar formation, which results in cleaner bio-oil and syngas with fewer impurities [30]. After the modification process, the modified mixture is cooled and stored properly to prevent any unwanted reactions or moisture uptake. The important reactions occurring during CaO catalytic biomass pyrolysis was shown in Table 2.

### 3.1. Physicochemical Properties of CaO-Modified Biochar

Typically, CaO-modified BC is a novel material created by treating standard BC with CaO, which significantly improves its physicochemical properties. This modification not only changes the structural attributes of BC, but also enhances its effectiveness in AD processes and agricultural uses. By increasing its surface area, porosity, pH, and functional groups, the modified BC becomes more efficient at stabilizing the AD of VBW and retaining nutrients. This leads to higher biogas production and nutrient-rich digestate, making it a valuable additive for enhancing soil quality and fertility. Several of these physicochemical properties are discussed in detail below.

#### 3.1.1. Porosity and Surface Area

The addition of CaO to BC greatly improves its porosity, leading to increased adsorption capacity and reactivity. Various studies have evidenced this relationship, highlighting the significance of CaO in optimizing the structural properties of BC. For example, Chen et al. [31] reported that modifying BC with CaO leads to increases in its porosity by mitigating agglomeration and improving oxygen release about 9% compared to conventional BC. This result is essential for optimizing BC’s surface area and adsorption properties. Specifically, the introduction of CaO helps maintain the porous structure of BC, allowing for greater accessibility of pollutants during the adsorption processes. Moreover, during the modification process, CaO reacts with the organic compounds present in BC, leading to the development of a more complex and interconnected pore structure. Li et al. [32] prepared (elm, fir, and bamboo) BC, followed by modification with CaO. The results showed that the functionalization of CaO with BC improves catalytic activity and provides active adsorption sites. This increased porosity allows for improved water retention and aeration in the soil, facilitating better root growth and microbial activity. Additionally, Hu et al. [33] noted that the increased pore volume offers a larger surface area for the adsorption of nutrients and contaminants. This makes CaO-modified BC particularly effective at retaining essential nutrients.

In addition, functionalization of BC with CaO remarkably increases its surface area, which is a crucial factor affecting its adsorption capacity and overall effectiveness in AD as an additive. This enhancement occurs through a combination of chemical reactions and physical changes during the modification process. According to Wang et al. [27], CaO promotes the formation of new pores and microstructures within the BC matrix, effectively expanding the surface area available for interaction with several components. Likewise, the rise in surface area provides more sites for microorganisms to attach and thrive, facilitating more efficient interactions during the breakdown of organic matter. As a result, microbial activity is elevated, leading to improved decomposition rates and higher biogas production [12]. Moreover, the larger surface area facilitates improved adsorption of pollutants, including ammonia nitrogen and other organic contaminants, making CaO-modified BC an effective material in the AD of VBW.

Furthermore, the functionalization of BC with CaO leads to significant improvements in both porosity and surface area, which are critical for enhancing its utility in agricultural and environmental applications such as AD. When CaO is added to BC, it undergoes a chemical reaction with moisture and carbon dioxide present in the environment, forming CaCO_3_ [27]. This process not only creates additional pore structures, but also expands the existing pore network within the BC matrix. Additionally, the effect of porosity and surface area of CaO-modified BC on the AD process is profound, remarkably increasing the efficiency of biogas production. Increased porosity allows for better retention of microbial communities, providing a more favorable habitat for anaerobic bacteria that are essential for breaking down organic matter [28]. This enhanced microbial colonization leads to improved degradation rates of substrates, resulting in higher biogas yields. Moreover, the increased surface area of the modified BC facilitates greater interaction between the BC and organic materials, promoting the adsorption of VFAs, ammonia and other intermediates produced during digestion [34]. This not only enhances the stability of the digestion process, but also reduces the inhibitory effects of certain compounds that can arise during the AD process.

#### 3.1.2. pH and Functional Groups

CaO plays a significant role in enhancing the alkalinity of BC, which can lead to increased biogas production during AD. When BC is enriched with CaO, it raises the pH level of the substrate, creating a more favorable environment for the microbial communities involved in biogas production. This elevated alkalinity helps to buffer the system against acidification, ensuring that the conditions remain optimal for the digestion process [28]. As a result, microbial activity is enhanced, leading to a more efficient breakdown of organic matter and subsequently higher yields of biogas. The presence of CaO not only promotes the growth of methanogenic bacteria, but also improves the overall stability of the AD treatment, making it a valuable additive for maximizing biogas production from organic waste. Moreover, the resultant higher pH in AD process enhances the properties of digestate, which can be employed to ameliorate acidic soils, making essential nutrients more available to plants [35]. Therefore, the application of CaO-modified BC leads to the increase in pH, which not only improves the BC’s effectiveness as an additive in AD processing of VBW to increase biogas production, but also improves the characteristics of digestate, which can be applied as organic fertilizer for soil amendment, contributing to healthier soil ecosystems and enhanced agricultural productivity.

Additionally, CaO-modified BC significantly enhances the production of biogas during AD by increasing the number of functional groups on its surface. This modification improves the BC’s chemical properties, making it more reactive and better suited for microbial colonization [36]. The increased functional groups provide additional sites for microbial attachment and activity, facilitating the breakdown of organic materials. During the modification process, CaO helps in the formation of new functional groups, such as carboxyl, hydroxyl, and carbonyl groups, which arise from the reaction between CaO and the organic matter in BC [27]. These additional functional groups increase the BC’s surface polarity and charge density, enhancing its ability to retain cations and anions, thereby improving its cation exchange capacity. This increased reactivity not only allows for better nutrient retention, making essential elements more available to methanogenic bacteria, but also enhances the BC’s capacity to adsorb pollutants like heavy metals, ammonia, and other organic contaminants [37]. Consequently, the augmentation of functional groups through CaO modification contributes to the overall efficacy of BC as an additive in AD, making it a valuable addition to waste management and renewable energy production strategies. The reaction between CaO and BC was simplified in Equation (4) to illustrate the formation of calcium-modified BC and the release of hydroxide ions. While BC is a complex mixture, the following chemical equation represents the interaction in a generalized manner.

BC + CaO + H_2_O → Ca-BC + 2OH^−^ (4)

where

- BC: Represents biochar, which contains various organic compounds and functional groups.
- CaO: Calcium oxide, which reacts with water to produce calcium hydroxide Ca(OH)_2_ in the presence of moisture.
- H_2_O: Water is often present in the environment, facilitating the reaction.
- Ca-BC: Calcium-modified biochar, indicating that calcium ions are now part of the biochar structure.
- 2OH^−^: Hydroxide ions released during the reaction, contributing to the increase in negative charge on the biochar surface.

This reaction illustrates how CaO can modify the surface chemistry of BC, potentially enhancing its properties for applications as an AD additive to increase the production of biogas. The aforementioned modification methods not only enhance the BC’s surface area, porosity, buffer capacity, adsorption, cation exchanges capacity, functional groups, and stability, but also promotes the generation of calcium-rich digestates, making it more beneficial for further applications, such as improvements to soil health and fertility [35]. Nonetheless, this is a basic depiction that does not capture all potential interactions or the precise stoichiometry, which would vary based on the specific type of BC, CaO used, and the conditions of the reaction.

## 4. Anaerobic Digestion of Vacuum Blackwater

### 4.1. Characteristics of Vacuum Blackwater

Typically, VBW is a specific type of wastewater generated from vacuum toilets, commonly found in marine vessels, trains, aircraft, and environmentally sustainable buildings [6]. Unlike traditional flush toilets, such as dual and conventional toilets that rely on large volumes of water to transport waste, vacuum toilets use air pressure to remove waste efficiently, resulting in a concentrated form of wastewater [38]. This method helps in water conservation and minimizes the volume of waste. The composition of VBW is distinct, comprising primarily human waste, urine, and toilet paper, along with small amounts of cleaning agents and personal care products. This wastewater is characterized by a high concentration of biodegradable organic matter, which makes it amenable to treatment through processes like AD. Additionally, VBW is rich in essential nutrients, such as N, P and K, which can be valuable for agricultural applications [39]. The concentrated nature of VBW allows for efficient digestion, which leads to higher biogas yields compared to more diluted blackwater streams [19]. Moreover, the AD of VBW contributes to sustainable waste management by reducing the volume of waste, minimizing environmental impacts, and promoting the recovery of renewable energy and nutrients, making it a compelling choice for resource recovery initiatives. Table 3 presents different characteristics of VBW compared with other backwater flush systems.

The physicochemical characteristics of VBW can vary depending on several factors, including the source of the blackwater, user behavior, retention time, seasonal variations, chemical additives, etc. It typically has a high organic matter content, reflected in its BOD and COD, indicating significant levels of biodegradable material. The pH of blackwater usually ranges from acidic to neutral [40], affecting microbial activity during treatment processes such as AD. Its density is higher than that of gray and brown water due to the concentration of suspended solids and organic compounds, which can lead to challenges in handling and processing [19]. Additionally, blackwater contains a variety of nutrients, including N, P, K, and other chemical substances, which if released untreated, can contribute to ecological problems [4]. The presence of pathogens, including bacteria and viruses, necessitates careful treatment to ensure safe disposal and prevent health risks [1]. The complexity properties of VBW highlights the need for innovative blackwater management solutions that can address both its environmental impact and resource recovery potential.

### 4.2. Anaerobic Digestion Process

Generally, AD is a biological process that breaks down organic matter in the absence of oxygen, resulting in the production of biogas and digestate [12]. This process is particularly effective for treating various types of organic waste, including food waste, municipal solid waste, agricultural residues, and wastewater. By harnessing the natural metabolic processes of microorganisms, AD not only reduces the volume of waste, but also converts it into valuable resources, such as renewable energy in the form of biogas, which can be used for heating, electricity generation, or as a vehicle fuel [7]. However, VBW poses unique challenges for waste management, as it contains high concentrations of organic matter, nutrients, and pathogens, making it difficult to treat using conventional methods [1,38]. As urbanization and population growth increase the demand for efficient waste management solutions, AD emerges as a sustainable and effective option for treating VBW, allowing for the recovery of energy and nutrients while minimizing environmental impacts. The AD of VBW offers several advantages. Firstly, it significantly reduces the volume of waste, decreasing the burden on treatment facilities and landfills [40]. Secondly, the biogas produced during the digestion process can be harnessed as a renewable energy source, contributing to energy sustainability and reducing greenhouse gas [41]. Additionally, the digestate, which is the residual material after digestion, can be processed further to create nutrient-rich fertilizers, thereby closing the nutrient loop and promoting sustainable agricultural practices [8]. Thus, AD presents a promising solution for managing VBW while simultaneously generating bioenergy and valuable byproducts.

However, AD of VBW, while beneficial, has several limitations that can impact its effectiveness and implementation. One major challenge is the high variability in the composition of VBW, which can lead to fluctuations in the microbial community and hinder optimal digestion conditions [39]. Moreover, the presence of inhibitors, such as ammonia, nitrogen, VFAs, and other toxic compounds, can adversely affect microbial activity and reduce biogas production [38]. The process also requires careful control of parameters such as temperature, pH, and retention time to ensure efficient digestion, which can complicate operational management. Because of the acidic property of VBW, it poses significant challenges to its AD process [40]. Typically, VBW can become acidic due to several interrelated processes: 1. the decomposition of organic matter, including human waste and other materials, leads to the production of VFAs as bacteria break down these substances, which contributes to a decrease in pH. 2. the breakdown of urea in urine forms ammonium ions (NH_4_^+^), which can further acidify the water when converted to NH_3_ under certain conditions, reacting with water to produce hydronium ions (H_3_O^+^), as shown in the chemical Equations (5) and (6).

(5) $${\left({\mathrm{NH}}_{2}\right)}_{2}\mathrm{CO}+H_{2}O\overset{Urease}{\to }2{{NH}_{4}}^{+}+{CO}_{2}$$

and

NH_4_^+^ + H_2_O ⇌ NH_3_ + H_3_O^+^ (6)

In addition, 3. the presence of sulfur compounds in VBW can also lead to the formation of hydrogen sulfide (H_2_S) and sulfuric acid (H_2_SO_4_), both of which contribute to the acidity during the AD process. Moreover, the inhibitory effects of ammonia and H_2_S in the AD are significant challenges that can hinder the efficiency of the digestion process. Ammonia, often present in high concentrations due to the breakdown of nitrogenous compounds, can lead to an increase in pH levels, which adversely affects the activity of methanogenic microorganisms. Elevated ammonia levels can also be toxic, inhibiting microbial growth and metabolic functions, ultimately reducing biogas production [38]. Similarly, H_2_S, a by-product of protein degradation and sulfate reduction, poses additional toxicity risks. It can inhibit key enzymes involved in the AD process and lead to the corrosion of equipment, complicating operational management [42]. The presence of these compounds can shift the microbial community’s dynamics, favoring sulfate-reducing bacteria over methanogens, further decreasing the overall efficiency of biogas production. Furthermore, 4. the microorganism’s activities lead to the generation of carbon dioxide, which dissolves in water to form carbonic acid (H_2_CO_3_), adding to the overall acidity. The aforementioned factors can lead to an acidic environment in the digester, significantly hindering the process of AD, which relies on a balanced microbial community to break down organic matter in the absence of oxygen. Hussain et al. [43] reported that when the pH drops below the optimal range, typically between 6.5 and 8.0, it can adversely affect the activity and growth of key microorganisms involved in the digestion process, particularly methanogens, which are responsible for converting organic acids into methane. Thus, CaO-modified BC presents a promising solution to mitigate the challenges associated with the AD of VBW by creating a more favorable environment for AD treatment and maximizing the potential for biogas and nutrient recovery.

## 5. Effects of Calcium Oxide Modified Biochar in Anaerobic Digestion of Vacuum Blackwater

In general, VBW refers to wastewater that contains human waste and is collected using a vacuum system [6]. This type of waste contains a high load of organic materials, which makes it suitable for AD treatment [19]. AD is a biological process that breaks down organic matter in the absence of oxygen, resulting in the production of biogas and digestate [38]. For effective biogas production from AD, it is essential to manage or treat inhibitory substances such as ammonia, VFAs, sulfides and other chemical compounds. This process offers significant potential for waste treatment because it can generate biogas from organic wastes in an ecological and economical manner by providing a valuable source of bioenergy, which can be used in the generation of heat, bio-fuel, or production of electricity, while the digestate can serve as a great source of bio-fertilizer to improve soil properties. Numerous studies have indicated that BC is an excellent additive in the AD process due to its ability to regulate pH; improve the cation exchange capacity; promote thermochemical conversion, reactor stabilization, inhibitor absorption, and the microbial habitat; and enhance nutrient recovery [11,12]. Zhang et al. [44] examined the impact of nine different types of BC produced from three distinct feedstocks on the AD of sewage sludge. The author found that adding a suitable amount of BC was advantageous for enhancing cumulative biogas yield, whereas excessive amounts could hinder the AD process. Furthermore, modified BC has demonstrated improved versions of the aforementioned properties [14,15].

CaO-modified BC was found to have various beneficial effects on the AD of VBW, which is heavily enriched with organic materials due to its source from vacuum toilets [6,38]. In addition, this modified BC not only eliminates inhibitors from AD, but also enhances production of biogas and produces Ca-rich digestate. CaO improves the structural properties and porosity of BC, facilitating better microbial colonization and activity. According to Zhiwei et al. [45], this modification increases the surface area available for microbial attachment and enhances the adsorption of nutrients, which leads to more efficient degradation of organic matter. Zhang et al. [34] studied the effectiveness of CaO in dry AD of kitchen wastes. The results indicated that CaO enhanced biogas production; at a dosage of 0.07 g/g, biogas production reached 656.84 mL/g of suspended solids, which is about 8.38 times higher than that of the control. Additionally, the alkaline nature of CaO helps to maintain optimal pH levels in the digester, promoting a favorable environment for methanogenic bacteria. Wang et al. [28] examined the influences of CaO on the co-digestion of excess sludge and plant waste in mesophilic anaerobic conditions to enhance biogas production. The results show that CaO markedly increased biogas yield in the co-digestion system, with an optimal addition of 6% leading to a peak production of biogas of 461 mL/g of volatile solids, approximately 1.3 times higher than control.

Moreover, Ruolin et al. [46] recommended pretreatment of rice straw using CaO by liquid fraction of digestate as environmentally and economically feasible technology for a high-quantity/quality of biogas production. The findings indicated that the optimum biogas yield achieved was 308 mL/g of volatile solids, which is 77.4% greater than the control. Liu et al. [36] applied CaO pretreatment as a novel technology to improve the performance of the AD of food waste. The findings indicated that the strategies significantly enhanced the solubilization of organic matter in food waste and boosted the hydrolysis rate of AD, leading to a higher biogas yield. The maximum produced biogas of 284.4 mL/g was achieved in the group treated with 1.0 g/L CaO, representing a 23.8% increase compared to the control group. Yang et al. [47] examined the positive effect of Ca addition in the AD process. The author concluded that the addition of Ca(OH)_2_ and CaCO_3_ positively influenced ryegrass biomass. It was found that higher concentrations of Ca had more pronounced effects. The addition of Ca-enhanced biogas production with a low concentration of CaCO_3_ yielded a highest biogas output of 372.2 mL/g VS, which was 61.7% higher than the control group. Conversely, a high concentration of Ca(OH)_2_ resulted in a maximum biogas production of 422.8 mL/g VS, an increase of 83.7% compared to control. Thus, the inclusion of CaO-modified BC in AD not only improves the overall microbial activity, but also accelerates the breakdown of organic materials, ultimately leading to increased biogas yields and a more stable and efficient digestion process.

In addition, CaO-modified BC has emerged as an effective adsorbent for inhibitors in AD treatment. The modification of BC with CaO enhances its surface properties, increasing its adsorption capacity for various organic and inorganic compounds that can hinder microbial activity during digestion. For instance, VFAs serve multiple functions in AD. They act as substrates for biogas generation and are crucial indicators for evaluating and managing the stability of the digestion process [28]. However, the buildup of VFAs can lower the pH of the reactor and hinder the process of CH_4_ production [48]. Thus, by tracking VFAs concentrations and the VFA/alkalinity ratio, operators can promptly adjust process parameters to maintain the efficient and stable functioning of the AD processes. In Zhang et al.’s [34] study, CaO effectively facilitated the biotransformation of VFAs, thus mitigating their adverse effects on biogas production. Anwar et al. [49] studied the role of CaO in sludge granulation and methanogenesis for the treatment of palm oil mill effluent, using up the flow in an anaerobic sludge blanket reactor. The results showed that the addition of 10 g of CaO per liter significantly reduced VFA concentration in the reactor.

Moreover, ammonia, a by-product of protein degradation, can accumulate and inhibit microbial activity in AD if not managed properly [38]. CaO-modified BC can act as an effective adsorbent for ammonia by forming calcium ammonium compounds, which helps to reduce free ammonia concentrations in the digester. Sun et al. [50] has studied the impact of metal ions, including calcium (Ca), magnesium (Mg), copper (Cu), zinc (Zn), and iron (Fe), on reducing ammonia inhibition during AD. The addition of Ca was deemed the most effective strategy, resulting in a 25% increase in biogas production by enhancing the activity of dehydrogenases and strengthening protein-binding structures. This adsorption process not only mitigates ammonia toxicity but also improves the overall efficiency of the digestion process by promoting a more favorable environment for microbial growth. Furthermore, the use of CaO can enhance the buffering capacity of the digestate, aiding in pH stabilization, which is crucial for optimal microbial performance [36]. By promoting a stable environment with lower concentrations of inhibitory substances, the modified biochar supports the growth of beneficial microorganisms that drive the AD process. Thus, the use of CaO-modified BC not only reduces ammonia and VFA levels, but also contributes to a more efficient and stable AD system.

**Figure 2:**
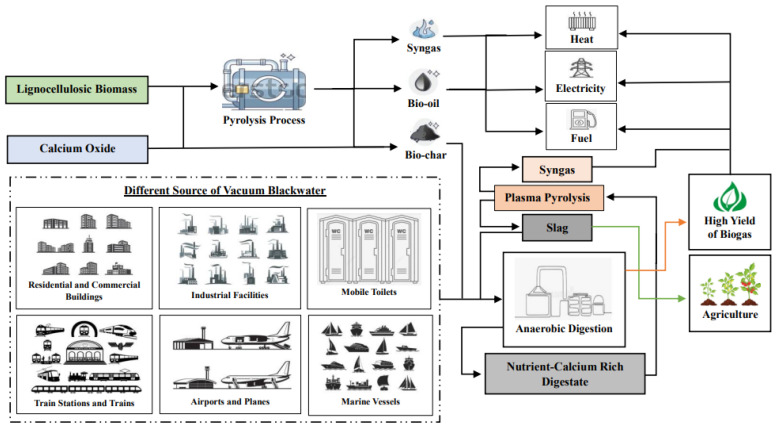
Schematic representation of calcium-oxide-modified biochar as an additive in anaerobic digestion of vacuum blackwater co-processed with plasma pyrolysis.

Moreover, the modification of BC with Cao significantly enhances nutrient retention and nutrient recyclability, making it a valuable amendment for agricultural soils. Tang et al. [51] studied the role of CaO for improving the phosphorus recycling method from sewage sludge. The results showed that the addition of CaO facilitated the development of hydroxylapatite (Ca_5_(PO_4_)_3_(OH), a beneficial P compound for plant growth. The modification process increases the surface area and porosity of BC, allowing it to better adsorb essential nutrients such as N, P, and K. Lee et al. [52] examined the removal of P from water using Ca-rich organic waste and assessed its potential as a fertilizer for rice cultivation. This study reported that the enhanced P adsorption capacity in mussel shell due to calcination was linked to the transformation of the mineral structure from CaCO_3_ to CaO and Ca(OH)_2_. This transformation of the mineral structure increased the release of Ca^2+^, which interacted with P to produce Ca_5_(PO_4_)_3_(OH). In addition, Zhang et al. [53] investigated how nitric oxide (NO) and nitrogen dioxide (NO_2_) interact with alkaline solids in the presence of water vapor found in flue gas at low temperatures. According to the Guo et al. [54] study, CaO will promote the fixation of nitrogen in digestate. The inclusion of an alkaline agent will significantly improve the overall ammonia recovery by up to 74% [37]. This improved nutrient retention not only helps to prevent leaching, which can occur in liquid waste streams like VBW, but also promotes more sustainable nutrient cycling within the soil ecosystem. As a result, crops can access these nutrients more efficiently, leading to improved growth and yield. Furthermore, the enhanced properties of CaO-modified BC used as an additive in the AD process will enhance nutrient recycling and generate a Ca-rich digestate, contributing to soil health by fostering beneficial microbial activity and improving soil properties, which are crucial for long-term agricultural productivity [35]. Thus, the use of proposed CaO-modified BC in AD not only improves nutrient retention, but also enhances the stability and efficiency of the digestion process, leading to increased biogas production and better waste management outcomes. Schematic representation of CaO-modified BC as an additive in AD of VBW co-processed with plasma pyrolysis was shown in Figure 2.

As shown in Figure 2, the application of CaO-modified BC as an additive in the AD of VBW, combined with the subsequent feeding of digestate into plasma pyrolysis, exemplifies a cutting-edge strategy for optimizing resource recovery and pathogen destruction. The modification of BC with CaO significantly enhances its alkalinity and adsorption characteristics, which are essential for boosting microbial activity and improving the breakdown of organic compounds during AD [34]. This process not only maximizes biogas production, but also stabilizes nutrients within the digestate, making it a valuable resource [47]. The subsequent plasma pyrolysis of digestate further ensures the effective thermal conversion of residual organic matter, leading to the destruction of pathogens and the generation of high-quality slag and syngas [55]. The resulting slag can be utilized as a soil amendment, contributing to improved soil fertility, moisture retention, and carbon sequestration, thereby promoting sustainable agricultural practices [56]. Additionally, biogas and syngas generated from AD and plasma pyrolysis represent valuable renewable energy sources that can be harnessed for various applications. Biogas, primarily composed of methane and carbon dioxide, can be utilized directly for heating, powering engines, or be converted into electricity [41]. Similarly, syngas, a mixture of hydrogen and carbon monoxide produced during plasma pyrolysis, can be used as a clean fuel for gas turbines or internal combustion engines, or further processed into liquid fuels through gas-to-liquid technologies [57]. The versatility of these gases not only contributes to energy generation, but also supports the transition towards sustainable energy systems by reducing reliance on fossil fuels. This integrated approach not only tackles the challenges associated with VBW management and pathogen control, but also facilitates the multiple uses of byproducts such as biogas, syngas, and slag. These byproducts enhance energy and food security, promote waste-to-energy solutions, and contribute to the reduction of greenhouse gas emissions. As a result, this method is a vital component of contemporary renewable energy strategies.

## 6. Techno-Economic Analysis of Proposed Technology

The incorporation of CaO-modified BC into the AD of VBW presents a promising avenue for enhancing the efficiency of the digestion process while simultaneously promoting the recovery of vital resources. AD, a biological process that breaks down organic matter in the absence of oxygen, is recognized for its ability to convert waste into biogas, which can be utilized as a renewable energy source. When combined with CaO-modified BC, the AD process can benefit from improved nutrient retention, enhanced microbial activity, and increased biogas production [28]. In addition to AD, plasma pyrolysis emerges as another prominent waste-to-energy technology [55]. This method utilizes high temperatures generated by plasma to decompose organic materials, resulting in syngas and BC, both of which can be harnessed for energy and resource recovery [58]. Understanding the techno-economic implications of these processes is crucial for their successful implementation in industrial settings. Insights into cost-effectiveness, operational efficiency, and environmental benefits will guide policymakers and investors in making informed decisions that support sustainable development goals. As the global demand for renewable energy and effective waste management solutions continues to rise, exploring innovative approaches like the use of CaO-modified BC in AD and co-processing with plasma pyrolysis will be essential for fostering a circular economy and promoting environmental sustainability.

Biochar has demonstrated the ability to decrease the levels of COD and VFAs, thereby enhancing production of methane in AD treatment. Jingran et al. [12] studied the effect of BC on the AD of swine manure inoculum (SMI) and cellulose–peptone–swine inoculum (CPSI). The findings indicated that methane yields from CPSI were 20.3% to 38.7% higher compared to those from SMI without BC. Biochar promotes decomposition rate of COD and mitigates potential ammonia inhibition through adsorption across various inoculum sources. Analyses of the microbial community revealed that the introduction of BC supports the proliferation of Clostridiales and Bacteroidetes, as well as increases the relative abundance of hydrogenotrophic methanogens, specifically *Methanobacterium* and *Methanobrevibacter*. In addition, Joisleen et al. [59] reported that a concentration of 6.67 g/L with BC reduced VFAs and COD by 42.75% and 88%, respectively, which led to an increase in methane yield of 18%. Additionally, Giwa et al. [55] combined AD with plasma pyrolysis, and their analysis revealed that co-processing of both methods offers several benefits in terms of bioenergy production, reduction of waste volume, adsorption of valuable material, and nutrient recovery. Furthermore, VBW is classified as hazardous waste due to its high content of pathogens, organic matter, and toxic substances. This characteristic makes VBW an ideal feedstock for co-processing, allowing for the comprehensive utilization of its contained materials. AD is noted for its environmental and economic advantages in treating VBW, as it effectively reduces VFAs and COD, facilitates nutrient recycling, diminishes pathogens, stabilizes microbial populations, and recovers carbon through biogas production [19]. However, the direct application of digestate in sectors such as agriculture is not recommended due to the potential accumulation of toxic chemical substances and infectious microorganisms, which may result in second pollution. Therefore, digestate can be directed to plasma pyrolysis, where it can be converted into syngas and slag. This process not only helps in managing the digestate safely, but also allows for the recovery of valuable energy and materials, contributing to a more sustainable waste management approach. By transforming the digestate into syngas, which can be used for energy production, and slag, which can serve as a soil conditioner or as an AD additive [60], co-processing of AD and plasma pyrolysis presents a zero-waste solution by effectively converting VBW into valuable resources.

However, the integration of these processes may lead to higher energy consumption, raising concerns regarding energy demand. These concerns can be mitigated by the energy produced, which has the potential to generate additional revenue when reintegrated into the processing facility. This approach not only enhances resource utilization for bioenergy production, but also facilitates nutrient recycling and provides a safe disposal method for large volumes of VBW in a manner that is both economically and environmentally sustainable. The economic assessment of using CaO-modified BC as an AD additive will hinge on several factors, including operating costs, revenue generation, production expenses, and sales prices. These financial metrics will vary based on the plant’s capacity and the costs associated with the byproducts of both AD and pyrolysis. A thorough evaluation will be essential to determine the overall feasibility and profitability of integrating CaO-modified BC into the AD process, taking into account the potential benefits and challenges associated with its implementation. Zhang et al. [41] studied the economic feasibility application of BC into AD. The results indicate that the addition of BC led to a total annual methane production of 1.01 × 10^8^ m^3^, compared to 7.39 × 10^7^ m^3^ without BC. This represents an increase of 26.83% in methane production relative to the control, which can be utilized for generating heat energy, fuel, and electricity. On the operational side, the annual costs for managing the plant were estimated to be between USD 4.06 million and USD 6.09 million. Meanwhile, the net profit generated from the additional methane produced was approximately USD 9.99 million, resulting in a total annual income ranging from USD 3.90 million to USD 5.93 million.

Moreover, the economic viability of plasma pyrolysis for medical waste treatment in Saudi Arabia was thoroughly examined by [58]. The study revealed that Makkah has 10,500 hospital beds, generating an average annual medical waste weight of 2,835,000 tons. This waste can yield approximately 2,268,000 tons of pyrolysis oil, producing energy equivalent to 90 billion megajoules. In the proposed plasma treatment initiative, the distillation process is expected to produce up to 1,928,000 tons of diesel oil, with projected electricity sales profits estimated at 21 million megawatt-hours. In 2022, the revenue from pyrolysis oil sales reached around USD 34.44 million, while the income from electricity generated using the diesel oil amounted to USD 1020 million for residential use and USD 1445 million for industrial applications. Furthermore, Ahmed et al. [61] reported that plasma gasification is the most environmentally friendly and economical method for treating plastic waste. In 2022, the process recovered approximately 317,000 tons of pyrolysis oil, yielding energy equivalent to 12.55 billion megajoules and achieving an output efficiency of 81%. The authors presented a roadmap outlining the economic and environmental objectives, indicating an economic return on investment of 80%, a payback period of 1.2 years, and a gross profit margin of 129%. Consequently, this comprehensive economic analysis highlights the significant financial potential of plasma pyrolysis, which could similarly apply to the plasma pyrolysis of digestates derived from the AD of VBW. Additionally, this review advocates for the incorporation of CaO-modified BC as an additive in the AD process of VBW to enhance biogas production and generate nutrient-rich digestate. Rather than being discarded, this digestate can be processed through plasma pyrolysis, converting it into syngas and slag. This approach not only optimizes resource recovery but also reduces environmental impact and generates additional revenue streams. Future research is essential to determine the exact financial viability of this recommended technology.

## 7. Challenges and Future Directions

Despite the promising benefits of using CaO-modified BC as an additive in the AD of VBW co-processed with plasma pyrolysis, several considerable challenges must be addressed to optimize its implementation. The operating temperature of the AD reactor is critical when using CaO-modified BC as a co-substrate in the digestion of VBW. This is largely due to the temperature sensitivity of both CaO and the microbial communities involved in the AD process [34]. Calcium oxide exhibits varying reactivity at different temperatures, which can influence its effectiveness at enhancing microbial activity and nutrient availability. At elevated temperatures, CaO can facilitate the breakdown of organic matter and improve the solubilization of nutrients, but excessive heat may also lead to the degradation of the BC structure, reducing its surface area and adsorption capacity [27]. Moreover, the microbial populations in the AD reactor are highly sensitive to temperature fluctuations. Optimal temperature ranges, typically between 35 °C and 55 °C, are necessary to maintain the activity of methanogenic bacteria, which play a crucial role in biogas production. If the temperature exceeds these optimal levels, it can inhibit microbial metabolism, leading to decreased biogas yields and potential disruptions to the digestion process [34]. Therefore, careful monitoring and control of the operating temperature are essential to ensure that the benefits of using CaO-modified BC are fully realized while maintaining the stability and efficiency of the AD process. Balancing these factors is key to optimizing the implementation of CaO-modified BC in AD systems for VBW treatment.

In addition, the preparation process for using CaO must be meticulously controlled; improper handling can lead to stability loss, diminishing its effectiveness as an AD additive. Variations in temperature, humidity, and mixing techniques during the modification process can significantly impact the chemical properties of the CaO-modified BC, affecting its performance in enhancing microbial activity and nutrient availability [27]. Furthermore, the optimal stoichiometric ratio for modifying BC with CaO requires thorough investigation, as an incorrect ratio can result in inadequate activation of the BC or excessive alkalinity, both of which could hinder the digestion process. Thus, comprehensive study is essential to identify the ideal conditions for preparing CaO-modified BC, ensuring its stability and efficacy in AD applications. While preliminary studies suggest that CaO-modified BC can improve results in the AD process [46,47], such as enhancing methane production and facilitating nutrient recovery, a comprehensive understanding of the specific mechanisms by which CaO affects AD systems remains insufficient. Moreover, the detailed effects and mechanisms by which CaO-modified BC influences pathogens in the AD process of VBW warrant thorough investigation. Due to this, modification could alter the surface charge and functional groups of the BC, facilitating stronger interactions with pathogens and promoting their removal or inactivation during the digestion process. Additionally, the alkaline nature of CaO may help to raise the pH of the BC, creating an unfavorable environment for many pathogens while simultaneously supporting beneficial microbial communities [62]. Evaluating these dynamics is crucial for optimizing the use of CaO-modified BC in AD, as it can improve pathogen control, enhance biogas production, and promote overall system efficiency. Further research will be essential to elucidate these mechanisms and quantify the effectiveness of this approach in managing pathogens within VBW treatment systems. Although CaO tends to adhere well to BC surfaces, this attachment may vary across different types of BC produced from diverse feedstocks, primarily due to variations in their physicochemical properties. Such inconsistencies can lead to variable modification efficiencies for each batch processed. Therefore, it is crucial to identify the most suitable pyrolysis operating conditions and feedstocks for producing BC that can be effectively modified with CaO, ensuring consistent performance and maximizing the benefits of this innovative approach in AD applications.

Additionally, the operating conditions of plasma pyrolysis are critical factors that significantly influence the quantity and quality of byproducts. It is essential to carefully evaluate the optimal operating conditions in relation to the digestate. Qing et al. [27] have indicated that calcium has a positive effect on the pyrolysis process. Therefore, future research should focus on the impact of calcium in plasma pyrolysis, particularly because calcium-rich digestate is recommended to be channeled into plasma pyrolysis systems following AD. A critical evaluation of how calcium influences plasma pyrolysis outcomes could enhance the efficiency and effectiveness of the process, potentially leading to improved energy recovery and resource management. Moreover, a comprehensive evaluation of the environmental implications associated with slag disposal is also necessary, particularly when it is used as a soil conditioner [55]. This is especially important given that VBW contains various known and unknown toxic chemical compounds and other harmful microorganisms, which may survive the operating temperatures of plasma pyrolysis. Therefore, understanding the interactions between the digestate and the plasma pyrolysis process is vital to ensure safe and effective utilization of the resulting slag, particularly after prolonged use. In summary, although there are some challenges, CaO demonstrates promising potential in the treatment of VBW through the co-processing of AD and plasma pyrolysis. However, optimization of the treatment process and additional comprehensive study are necessary to fully understand its benefits and expand its uses across various applications. Consequently, while CaO-modified BC enhances AD performance and promotes resource recovery, it is essential to further investigate the interactions among properties of BC and microbial community dynamics. Such study will be crucial for optimizing the process and promoting a high yield of AD byproducts. By addressing these factors, we can better harness the benefits of CaO-modified BC in sustainable waste management solutions.

## 8. Conclusions

The use of CaO-modified BC in the AD of VBW shows significant potential for increasing biogas production and recycling valuable nutrients. By enhancing the physicochemical properties of BC, CaO modification improves its adsorption capacity and reactivity, which facilitates better interactions with the microbial communities involved in the digestion process. This enhancement can lead to increased methane yields and a more efficient breakdown of organic matter. Additionally, incorporating CaO-modified BC can help regulate pH, mitigate inhibitors, and stabilize nutrients, making them more accessible for microbial uptake while reducing nutrient leaching in the digestate. The digestate from AD can be directed into plasma pyrolysis to produce syngas and slag, providing a valuable pathway for resource recovery. Both biogas and syngas generated during AD and plasma pyrolysis, respectively, can be utilized as renewable energy sources. The resulting slag can serve multiple purposes, particularly as a biofertilizer or as an additive in the AD process itself, enriching the substrate with essential nutrients and enhancing microbial activity. This dual-use strategy not only maximizes the value derived from the digestate, but also contributes to a circular economy by promoting sustainable agricultural practices. Consequently, this innovative approach enhances the overall efficiency of VBW treatment while supporting sustainable waste management practices through resource recovery and minimizing environmental impacts.

## Figures and Tables

**Table 1:** Physiochemical properties (pH, pore volume, specific surface area, volatile matter, ash content, product yield and total carbon content) of biochar produced from different types of feedstocks at various temperatures and residence times.

Substrate	Pyrolysis Condition		pH	Pore Volume (cm^3^/g)	Specific Surface Area (m^2^/g)	Volatile Matter (%)	Ash Content (%)	Product Yield (%)	Total Carbon Content (%)	Reference
	Temperature (°C)	Residence Time (min)								
Corn Stover	300	120	7.70 ± 0.08	0.00843	-	54.0 ± 0.6	5.7 ± 0.2	66 ± 2	45.5 ± 0.5	[21]
Corn Straw	600	60	10.4	0.012	7	-	18	26.7	58.6	[22]
Soybean Stover	300	180	7.76 ± 0.06	-	5.61	46.34 ± 2.99	10.41 ± 0.5	37.03	68.81	[10]
Wheat Straw	600	180	9.1	0.091	183.3	-	8.11	33.4	72.9	[24]
Corn Stover	500	120	9.7 ± 0.005	0.00722	-	33.8 ± 0.5	18.7 ± 0.3	29.2 ± 0.3	64.5 ± 1	[21]
Peanut Shell	700	60	9.9	0.033	49	-	12	25.8	74.4	[22]
Peanut Shell	700	180	10.57 ± 0.05	0.20	448.2	32.65 ± 0.74	8.91 ± 0.08	21.89 ± 2	83.76	[10]
Herb Residue	600	180		0.051	51.3	-	31.1	31.2	55.0	[23]
Corn Stover	400	120	8.80 ± 0.20	0.00689	-	45.5 ± 0.4	12.5 ± 0.2	37 ± 1	64 ± 1	[21]
Wheat Straw	500	60	8.3	0.090	111	-	11	27.6	70.3	[22]
Peanut Shell	300	180	7.27 ± 0.03	-	5.61	60.47 ± 5.43	1.24 ± 0.08	36.91 ± 2	50–60	[10]
Rice Straw	600	180	9.7	0.084	156.2	-	10.65	34.3	78.5	[24]
Soybean Stover	700	180	11.32 ± 0.02	0.19	420.3	14.66 ± 1.68	17.18 ± 0.2	21.59 ± 1	81.98	[10]
Paper Mill Sludge	700	60	9.37	0.083	67.0	15.74	56.13	32.48	25.66	[25]

**Table 2:** Important reactions occurring during CaO catalytic biomass pyrolysis [27].

Name of Reaction	Chemical Equation
Pyrolysis	$\;\mathrm{Biomass}+\mathrm{Heat}\;\overset{CaO}{\to }Gas\left(H_{2}+CO+{CO}_{2}+H_{2}O+{CH}_{4}+C_{n}H_{m}\right)$
Water Gas (primary)	C + H_2_O → CO + H_2_
Water Gas (secondary)	C + 2H_2_O → CO_2_ + 2H_2_
Tar Reforming	$Tar+H_{2}O\overset{CaO}{\to }H_{2}+{CO}_{2}+CO+\;\mathrm{hydrocarbons}$
Carbonation	CaO + CO_2_ → CaCO_3_

**Table 3:** Characteristics of vacuum blackwater compared with other sources of backwater [38,39,40].

Properties	Units	Vacuum Toilet	Dual Toilet	Conventional Toilet
pH	-	6.7–8.6	8.5	8.4
VS	mg/L	14,200	2825	1847
TS	mg/L	17,140	3570	2390
TP	mg/L	202–330	70.5	38
TN	mg/L	1500–1700	410	190
TAN	mg/L	1040–1100	182	94.4
COD	mg/L	11,556–19,320	3105–4600	1544–2600
BOD	mg/L	5772	-	-
FVAs	mg/L	222	75	79
Free Ammonia	mg/L	355 ± 10.3	53 ± 1.2	24 ± 0.9

Potential hydrogen (pH); volatile solid (VS); total solid (TS); total phosphorus (TP); total nitrogen (TN); total ammonia nitrogen (TAN); chemical oxygen demand (COD); biochemical oxygen demand (BOD); free volatile fatty acids (FVAs).

## Data Availability

All data generated or analyzed during this study are included in this manuscript.
